# Isolated Cerebral Varix in A Case of Schizophrenia

**DOI:** 10.1192/j.eurpsy.2026.11629

**Published:** 2026-07-31

**Authors:** L. A. Bela, K. J. K. Zimri

**Affiliations:** Department of Psychiatry, Fauji Foundation Hospital, Rawalpindi, Pakistan

## Abstract

**Introduction:**

Cerebral varices are venous anomalies in the brain, usually associated with vascular abnormalities such as developmental venous anomalies or arteriovenous malformations, but may be found as isolated lesions as well (Erok B et al. PJR. 2022 Jun 27;32). Isolated lesions are a rare entity and therefore difficult to diagnose. They can also be misdiagnosed as space-occupying lesions in the brain (Tan ZG et al. Medicine. 2016 Jun 1;95(26):e4047).

Schizophrenia is a morbid mental illness characterized by disorders of thought, behavior and perception affecting a patient’s contact with reality. This disorder can manifest with a broad range of symptoms, such as hallucinations, delusions, formal thought disorder, social withdrawal, self-neglect, and impaired social functioning.(Keshavan MS et al. Neuroimaging Clinics. 2020 Feb 1;30(1):73-83).

**Objectives:**

The case presented here depicts the co-existence of a rare phenomena: a 0.6 to 0.7cm isolated cerebral varix in a patient of schizophrenia.

**Methods:**

A right-handed 16-year-old female, with no relevant past or family history of neuropsychiatric illness, presented with a history of suspiciousness, self-muttering, hearing voices, decreased sleep, social withdrawal and self-neglect for past 20 days.

Neurological examination was normal. No focal neurological deficit was seen. On mental state assessment, speech was irrelevant and incoherent. Mood was fluctuating. Affect was inappropriate. Thoughts included overvalued idea of her aunt being against her. Loosening of association was noted. Patient was exhibiting hallucinatory behavior (gesturing and self-muttering). Second and third person auditory hallucinations were noted. Content of the voices was derogatory and the patient was distressed by them.

Her Magnetic Resonance Imaging (MRI) brain with contrast was performed to rule out organic pathology. It revealed cerebral varices over the left parietal lobe. Computed tomography (CT) angiogram and venogram brain were performed, revealing dilated left vein of Trolard (0.7 cm), signifying cerebral varix (Figures 1 and 2). The patient was diagnosed as a case of isolated cerebral varix. Neurology and neurosurgery department said that no intervention was needed and it was labelled an incidental finding.

**Results:**

Patient started improving after clozapine therapy. Frequency and severity of hallucinations decreased. Weekly follow ups showed sustained improvement.

**Image 1:**
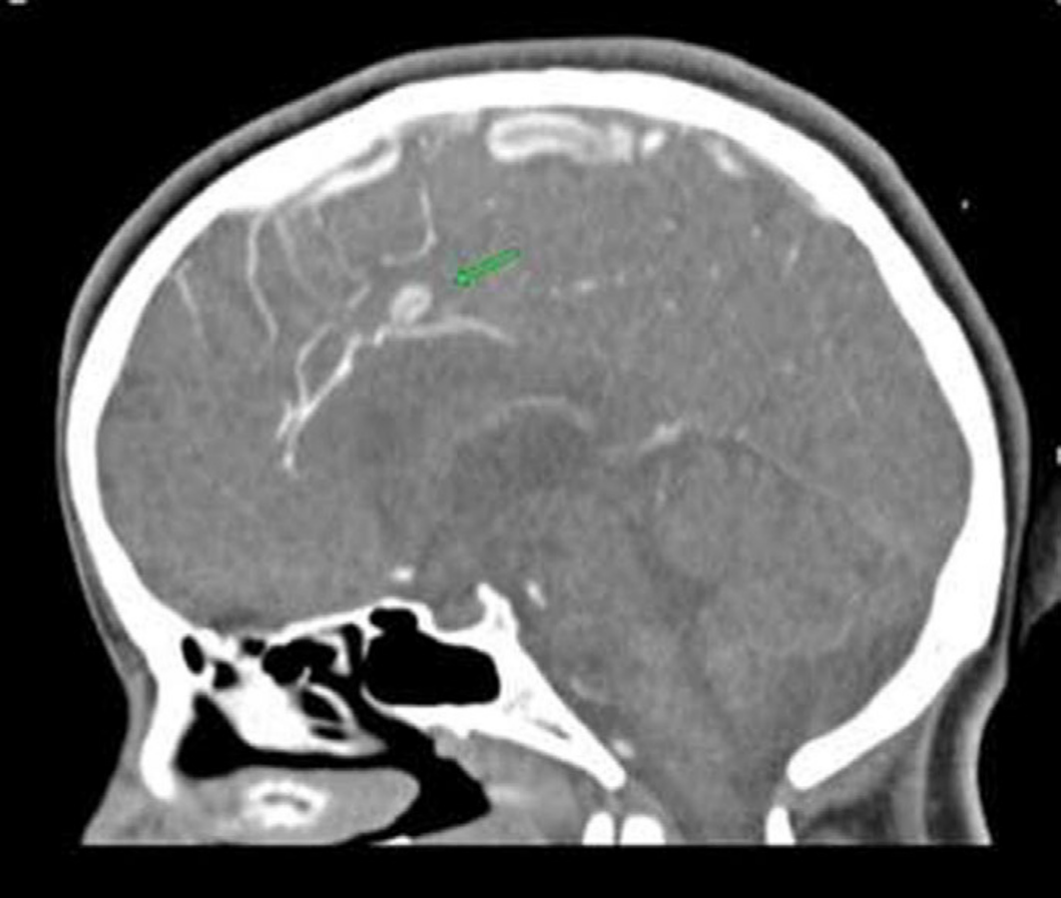

**Image 2:**
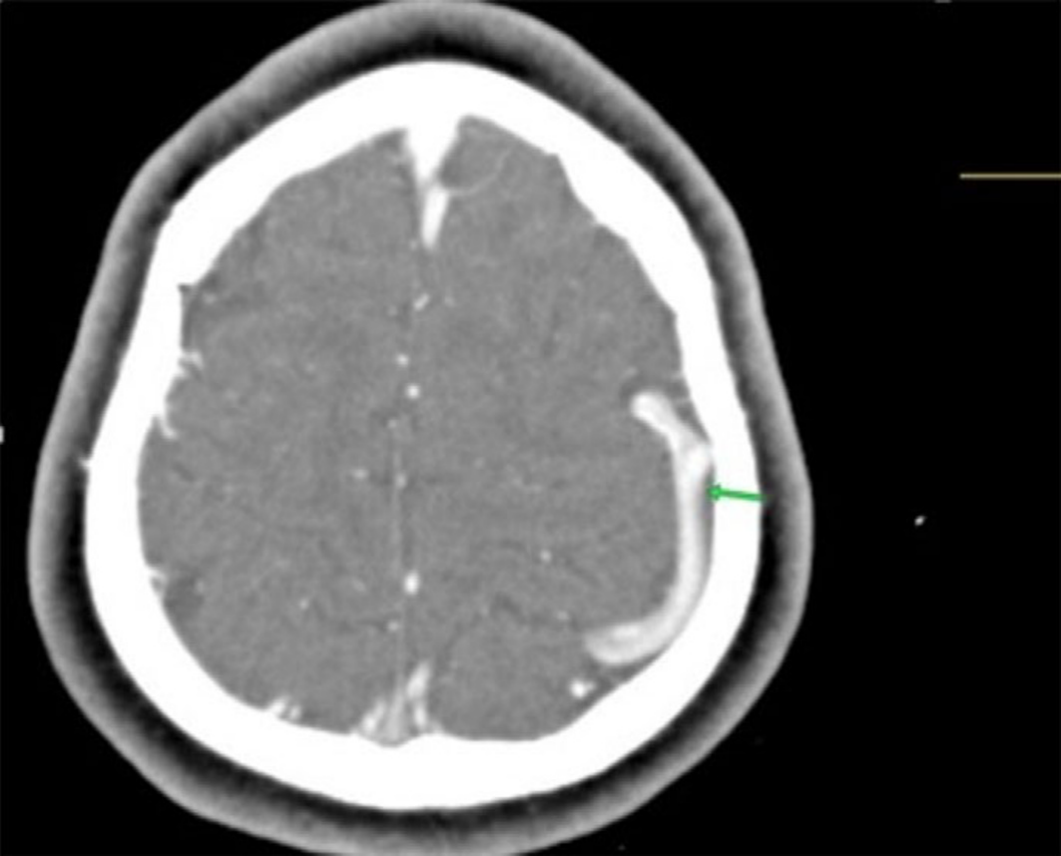

**Conclusions:**

Given the rarity of isolated cerebral varix and the complexity of schizophrenia, here we reported an unusual presentation of schizophrenia coupled with an unexpected finding of cerebral varix. The aim of documenting this report is to call attention to the importance of neurological examination and imaging in psychiatric patients.

**Disclosure of Interest:**

None Declared

